# Mitral Valve Replacement and Subaortic Membrane Resection Following Pneumonectomy

**DOI:** 10.1155/2010/480703

**Published:** 2010-03-02

**Authors:** Melih Hulusi Us, Murat Ugurlucan, Murat Basaran, Ozer Selimoglu, Ali Kocailik

**Affiliations:** Department of Cardiovascular Surgery, Goztepe Safak Hospital, Istanbul 34000, Turkey

## Abstract

The pulmonary status is a vital factor for patients undergoing open heart surgery. The cardiac surgery itself deteriorates the actual pulmonary functions. Today, patients are no longer living with a cardiac disease due to compromised respiratory functions secondary to various pathologies, patients with lung disorders more often seek solutions for their cardiac disease and they are commonly operated. However, the resection of a lobe or a whole lung is a major challenge for the patients planned for cardiac surgery. In this report, we present a 65-year-old patient, who had left pnemonectomy which had been performed 8 years ago and was admitted for mitral valve replacement and subaortic membrane resection.

## 1. Introduction

Open heart surgery is associated with postoperative deteriorated respiratory functions. Cardiac surgery is a challenge both for the patients and the whole cardiac surgery and reanimation team in the pre- and postoperative periods as well as during the surgery. The medical literature contains series on concomitant heart and lung surgery [1, 2]; however, it lacks a defined consensus for patients who have previously undergone pulmonary resection and who are planned for a cardiac surgery [2–7]. 

This report presents a 65-year-old male patient with 8 years history of pneumonectomy and who was operated for left ventricular outflow tract stenosis and mitral valve disease. 

## 2. Case Report

**Figure 1:**
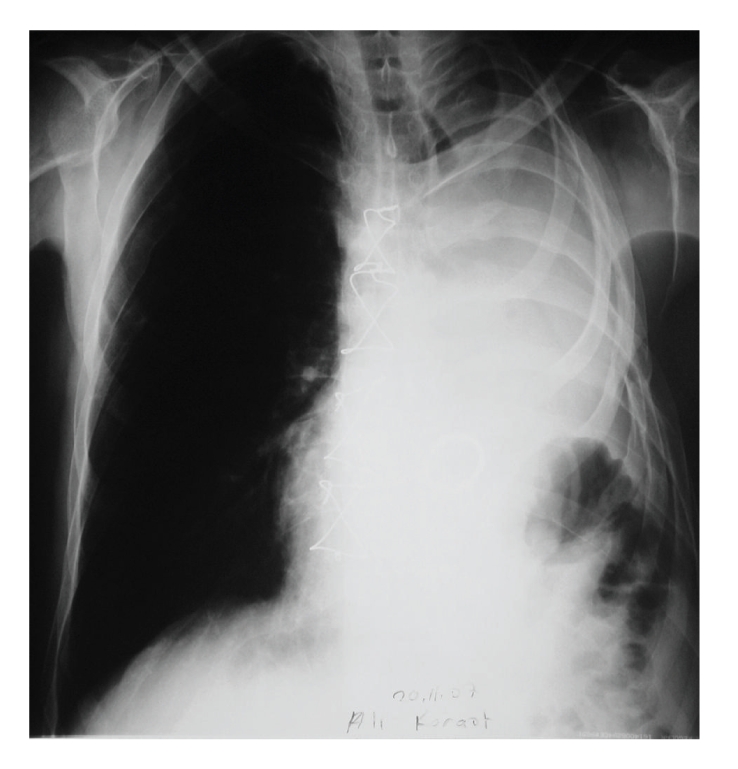
Chest roentgenogram of the patient postoperatively. Note the hyperinflated right lung with leftward displacement of the mediastinal structures.

A 65-year-old male patient presented to the clinic with dyspnea and easy fatigability. In his medical record there was a left pneumonectomy which was performed 8 years ago. The physical examination revealed normal temperature, blood pressure but atrial fibrillation. There was mid-late systolic murmur on cardiac auscultation. No respiratory sounds could be heard in the left hemithorax and right side lung sounds were normal. The chest X-ray examination indicated deviation of the mediastinal structures to the left and hyperinflation of the right lung (Figure 1). On echocardiography, there were severe mitral regurgitation and mitral stenosis most probably due to rheumatic origin (mean gradient of 10 mmHg with mitral valve area of 1.7 cm^2^), dilated left atrium (7.8 × 8.5 cm diameters), moderate tricuspid valve insufficiency (2-3+), and mild aortic regurgitation and a subaortic ridge with a mean aortic gradient of 25 mmHg, and ejection fraction of 55%. The pulmonary function tests showed a mixed obstructive and restrictive pattern with forced expiratory volume in 1 second and forced expiratory volume at 1 second/forced vital capacity of 1.30 L (45% of the predicted value) and 0.60 (50% of the predicted value), respectively. The room air arterial blood gas analysis revealed PaO_2_ of 60 mmHg, PaCO_2_ of 48 mmHg and oxygen saturation of 90%. After a week of pulmonary rehabilitation with chest physiotherapy, steroids, and bronchodilatators [4], the patient was scheduled for mitral valve replacement and subaortic membrane resection. 

Following standard median sternotomy, aortic, and bicaval cannulations, cardiac arrest was provided with antegrade cold blood cardioplegia. Topical ice slush was avoided to minimize the risk of phrenic nerve injury. The mitral valve was replaced with transseptal approach with a 29 mm St. Jude Medical Mechanical Heart Valve (St. Jude Medical, Inc.; St. Paul, Minn, USA). The subaortic fibromuscular membrane together with septal muscular resection was performed through aortotomy. Cross clamp and cardiopulmonary bypass times were 52 and 70 minutes, respectively. The patient was weaned off cardiopulmonary bypass with negative total fluid balance and on 5 *μ*gr/kg/minute of dobutamine and 3 *μ*gr/kg/minute of dopamine. At the intensive care unit, forced diuresis was achieved with furosemide to avoid pulmonary edema. Intravenous morphine sulphate was applied near extubation to minimize the pain induced respiratory dysfunction and the patient was extubated at the 6th postoperative hour. The inotropes were ceased on day 1 after the patient was discharged to the ward. The chest physiotherapy, steroids, and bronchodilatators were continued until the discharge on the 7th postoperative day. The patient has been regularly followed at our institution since the operation and is free off symptoms 6 months postoperatively. 

## 3. Discussion

The preoperative respiratory reserve is an important prognostic factor for patients undergoing open cardiac surgery; apart such procedures themselves are associated with respiratory compromise, atelectasis, and infections [1–5]. Except the emergent cases, cardiac surgery is planned in patients with optimal pulmonary conditions after lung rehabilitation therapy which may be performed according to the variable institutional pulmonology protocols [4]. However, it may not be possible in every case to achieve the optimal conditions prior to surgery. Chronic obstructive pulmonary patterns or lung cancer requiring surgery are frequently encountered in patients with heart problems. These patients are managed concomitantly during cardiac surgery or afterwards [1, 2]. On the other hand, patients with previous pulmonary operations and more extreme patients such as patients with pneumonectomy constitute a very small fraction of open heart operation candidates [3–7]. 

A number of issues have been raised in patients undergoing cardiac surgery with prior pneumonectomy. These mainly include maneuvers to prevent decreasing the present pulmonary status. The avoidance of fluid overload preoperatively is of utmost important in these patients with borderline respiratory reserve as well as taking precautions in order to protect the phrenic nerve during the mediastinal dissection and the internal thoracic artery harvesting in case of coronary artery bypass procedures [2–7]. We avoided topical cold ice slush application. The chest rehabilitation therapy with steroids in addition to bronchodilatators may contribute to the decrease in the inflammatory response to cardiopulmonary bypass by reducing the proinflammatory cytokines and increasing the interleukin-10 levels [8]. 

In a recent review by Stoller et al. [7], a series of 19 patients undergoing cardiac surgery with prior pneumonectomy included 4 patients who had valve replacement. In the series, the most common perioperative complication was pneumothorax [7]. In our patient, we preferred the left internal jugular vein for insertion of the pulmonary artery catheter to avoid a possible pneumothorax at the non-pneumonectomy side. 

As the exercise capacity of a patient with prior pneumonectomy and mitral regurgitation is poor, the patient is early symptomatic even when the regurgitation is not severe and the conventional cardiac surgery guidelines may need to be adapted on an individual basis [7]. The exposure of the heart in these patients is another challenge due to the leftward displacement of the mediastinal structures. In addition, the base of the heart is usually displaced posteriorly, hence the mitral valve annulus is difficult to reach through either transseptal or thansatrial approaches [4]. In our patient, we preferred a transseptal approach which turned out to be an efficient route. 

The accurate understanding of the risk factors and the outcome of the cardiac surgery, valvular surgery in particular, following prior pneumonectomy, have not been possible due to the very limited number of patients presented in the literature. In the review including both coronary artery bypass grafting and valve surgery patients by Stoller et al. [7], the overall mortality rate was 16% and well-exceeded valvular surgery in patients with preserved pulmonary functions. Only 5 to 8% of the mortality rate was attributed to the pulmonary complications. However, the perioperative respiratory failure was as high as 25% in the series comprising both coronary artery bypass grafting and valvular operations [7]. 

The surgical challenges in this group of patients in addition to high mortality/morbidity rates do not, however, preclude surgery. In appropriately selected cases, careful preoperative preparation aids in a favorable outcome. Short bypass times and short mechanical ventilation positively contributes to respiratory functions and prognosis.
